# Latent Class Analysis of Decompression Sickness Symptoms of Women Divers

**DOI:** 10.3390/healthcare10071246

**Published:** 2022-07-04

**Authors:** Da-Jung Kim, Jeong-Won Han

**Affiliations:** College of Nursing Science, Kyung Hee University, Seoul 02447, Korea; jung-321@hanmail.net

**Keywords:** decompression sickness, divers, women

## Abstract

This study aimed to investigate the types of clinical manifestations of decompression sickness among women divers (haenyeos) in Jeju using latent class analysis and to identify factors related to the condition. A total of 527 haenyeos who received their certification in diving fishery from Jeju and were working from 15 March to 31 May 2021 were included in this study. According to the results of the study, the latent classes were classified into type 1, type 2, and mixed symptoms groups (Akaike information criterion (AIC) = 6587.29, Bayesian information criterion (BIC) = 6698.23, sample size-adjusted BIC (saBIC) = 6615.70). For personal characteristics, age (χ^2^ = 40.31, *p* < 0.001) and education level (χ^2^ = 28.15, *p* < 0.001) showed a significant difference by latent class type. For work-related characteristics, diving experience (χ^2^ = 29.99, *p* < 0.001) and break time (χ^2^ = 9.32, *p* = 0.011) showed a significant difference by latent class type. The health-related characteristics, menopausal period (χ^2^ = 40.10, *p* < 0.001), body mass index (χ^2^ = 14.80, *p* = 0.013), and fatigue level (χ^2^ = 58.23, *p* < 0.001), showed a significant difference by latent class type. Rather than approaching the management of work-related diseases simply from the work environment perspective, it is important to increase the availability of health professionals who are capable of continuous health monitoring and management of women divers in their workplace.

## 1. Introduction

As of 2018, there were a total of 3820 haenyeos in Jeju, and the ages were very diverse. In general, haenyeos can dive from 50 s to 2 min, and the depth of the dive is about 7 m to 20 m. The workload of a haenyeo varies according to their diving ability [1]. Among the different types of divers, haenyeos refer to professional women divers who dive into the sea by implementing breath holds without any mechanical equipment to collect seafood [1,2]. In their typical diving practices, haenyeos stay in the water for a total of 155–341 min a day and complete 15–31 dives per hour [2]. A particular case that is much less frequent and less well known from a pathophysiological point of view is breath-hold diving-related decompression sickness (DCS) symptoms. Breath-hold divers do not breathe pressurized air, and the only inert gas they take in is the nitrogen that remains in the lungs from the last breath before the dive. Thus, the DCS reported in the literature in pearl and ama divers has been attributed to this progressive accumulation of residual nitrogen that increases with repetitive dives until it reaches critical oversaturation, which allows the formation of bubbles [3]. Divers absorb inert gas (nitrogen when air breathing) into tissues when breathing compressed gas during a dive, with more gas absorbed on deeper or longer dives. Even small intravascular bubbles may have physical effects, with inflammatory and thrombogenic host responses [4].

Decompression sickness is the most common effect based on symptomatology [3]. In addition, arterial gas embolism (AGE) can cause serious neurological manifestations. Recently, because of concerns that it may be difficult to distinguish clinically between DCS and AGE in some cases, the collective term DCI has often been used in clinical studies [5]. DCS can be classified into two types. Type 1 DCS is usually characterized by symptoms in the musculoskeletal system, skin, and fingers. Type 2 symptoms typically fall into three categories of neurological, inner ear, and cardiopulmonary symptoms [3]. The clinical manifestations of type 1 DCS may be seen within 24 h after diving in 98.0% of cases, but they tend to show quick recovery, whereas those of type 2 DCS are delayed and may lead to permanent damage [6]. The treatments for DCS can include normobaric oxygen therapy, hyperbaric oxygen (HBO) therapy, and high-flow oxygen therapy [7]. In Korea, HBO therapy is performed on haenyeos to treat DCS, but the number of haenyeos using HBO therapy has gradually decreased. The reason for the low frequency of use of HBO therapy is the problem of access to medical institutions. Moreover, HBO therapy is limited in the case of lung, cardiovascular, and ear diseases based on a past or current medical history. Because most haenyeos have these underlying diseases, treatment is limited. In most cases, haenyeos have restrictions regarding HBO therapy as a treatment option since it is known to potentially cause changes in vision such as nearsightedness, pain in the teeth or lungs, substernal burning, cough, and other symptoms, leading to its discontinuation [8]. Other possible treatment methods for DCS include administration of corticosteroids, lidocaine, tenoxicam, and diuretics [9] but the pressure generated during diving lowers the effect of the medication, limiting the effectiveness of pharmacotherapy [10].

DCS is one of the work-related diseases among the haenyeos of Jeju, South Korea, being caused by complex interactions among multiple factors. The determinant model of workplace productivity divides the causes of work-related diseases into job- and work-related factors, personal factors, and health and physical factors [11,12]. Through the determinant model of workplace productivity, the DCS of Jeju haenyeos can also be caused by the interaction of work-related factors, personal factors, and health-related factors. Therefore, the analysis of DCS symptom-related factors and their management is expected to be useful in the overall management of DCS. DCS-related risk factors reported from previous studies include deep diving depth [13] and longer diving experience [3]. It has also been reported that the diving skill acquisition path [14], number of rapid ascents [15], and break time [16] in diving practices are associated with the onset of DCS. In addition, the risk of DCS increases with increasing age, lower personal income, and education level [17]. Moreover, divers with various conditions, particularly respiratory diseases [18] and cardiovascular diseases, have a higher risk of hypoxia and air embolism, which also increases the risk of DCS [19]. Hormonal and metabolic changes are also associated with DCS, and divers who have experienced menopause have a higher incidence of these symptoms [20]; similarly, obesity [21] and fatigue level [22] are known to be associated with the occurrence of DCS.

To date, previous studies on DCS have mainly analyzed sports and commercial/fishery divers using underwater breathing apparatus when diving, with Jeju haenyeos included as one of the target samples for these studies. In particular, the DCS of Jeju haenyeos has different pathophysiological aspects from general DCS [3,4]. However, since most divers assessed in previous studies comprised those using underwater breathing apparatus, there are limitations in understanding the DCS of Jeju haenyeos from these existing studies [23]. In addition, in clinical practice, the diagnosis of DCS involves only its classification into type 1 and type 2, with few studies on the evaluation of a detailed status of DCS symptoms, which are often based on patients’ subjective complaints [3,24]. In particular, previous studies on the management of DCS symptoms in Jeju haenyeos mainly focused on drugs such as analgesics [25] and hyperbaric oxygen therapy [26], with research being limited to muscular skeletal diseases and headaches [27,28].

## 2. Materials and Methods

### 2.1. Study Design and Participants

This was a descriptive cross-sectional survey study to identify the types of clinical symptoms of DCS in Jeju haenyeos using latent class analysis, as well as investigate the factors related to DCS symptom types. Participants of this study included haenyeos who performed professional diving with a fishery diver certificate from the Jeju Special Self-Governing Province pursuant to Article 47(1) of the Fisheries Act. Those who complained of one or more of the detailed symptoms of DCS, without cognitive impairment, understood the purpose of the study, and gave their consent to participate were selected. 

### 2.2. Decompression Sickness (DCS)

For the analysis of DCS symptoms, according to the symptoms and types presented by Jain [29] and including the finger edema reported by Cha [23], a classification table was modified and supplemented to be used in this study. The level of severity for each symptom was measured on an 11-point Likert scale, and the score ranged from 0 to 10 for each item, with a higher score indicating a higher severity of the symptom.

### 2.3. Measurements

#### 2.3.1. Diving Class

The diving class [30] of haenyeos was measured and classified into the upper-skilled class, middle-skilled class, and lower-skilled class according to the criteria of the Jeju Special Self-Governing Province, which categorizes the class according to the diving depth and length of experience of diving of haenyeos.

#### 2.3.2. Diving Experience

The total period (unit: years) during which the study participants were engaged in catching and collecting fishery products was measured.

#### 2.3.3. Diving Skill Acquisition Path

The path through which the study participants first learned diving skills for fishery was classified into educational institution, senior haenyeo, and other (non-haenyeo), as indicated in the survey.

#### 2.3.4. Diving-Related Education

This item assessed whether the haenyeos had any experience of diving-related education for fishery diving.

#### 2.3.5. Rapid Ascent Experience

The experience of rapid ascent was assessed according to the criteria defined in Diver Healthcare Guidelines of the Korea Occupational Safety and Health Agency [31]. The defined criteria included risks from underwater organisms during one dive, malfunctioning of diving equipment such as buoyancy control device, problems with co-diving haenyeos, and cases of ascending without staying underwater due to unexpected changes in the underwater environment.

#### 2.3.6. Surface Break Time

The average time (unit: min) that the participants stayed on the sea surface during one dive was measured.

#### 2.3.7. Break Time

The average time (unit: min) that the haenyeos spent on board or outside the water for breaks during one dive was measured.

#### 2.3.8. Medical History

Items of medical history to be considered for diving according to the Diver Healthcare Guidelines of the Korea Occupational Safety and Health Agency [31] included participants’ status of diagnosis by physicians within the last 2 years, with respect to the following conditions: neurological disease, mental disease, cardiovascular disease, respiratory disease, otolaryngologic disease, ophthalmic diseases, endocrinopathy, gastrointestinal diseases, and other diseases.

#### 2.3.9. Menopausal Period

The menopausal period (unit: years) of the participants was measured.

#### 2.3.10. Body Mass Index (BMI)

The participants’ height and body weight were directly measured by the researcher using a digital weight and measuring station (CAS HC-1500, Korea), and the body weight (kg) was divided by the square of the height (m^2^) to calculate the BMI. According to the Guideline for the Management of Obesity in Korea by the Korean Society for the Study of Obesity [32], a BMI of 18.5–22.9 kg/m^2^ was classified as normal, 23.0–24.9 kg/m^2^ was classified as the pre-obesity stage, and 25.0 kg/m^2^ or higher was classified as the obesity stage.

#### 2.3.11. Fatigue Level

The fatigue level was measured before diving using a numeric rating scale (NRS), and the score ranged from 0 to 10, with a higher score indicating a higher fatigue level. The values were classified into mild (0–3 points), moderate (4–6 points), and severe (7 or more points) [33].

### 2.4. Data Collection

The survey data were collected from 15 March to 31 May 2021. The questionnaires of 527 subjects were analyzed in the final analysis. There is no established program or recommended standard for calculating the appropriate sample size for latent profile analysis. However, in a previous study [34] that performed a simulation on the determination of the optimal number of samples for latent class analysis, the type I error rate, power, and goodness-of-fit index were examined with different sample sizes of 200, 500, and 1000. The result showed that compared to the sample size of 200, sizes of 500 or 1000 were more suitable for the latent class analysis. In addition, although there are differences in the required sample size depending on the structure of the latent profile model, such as the number of sub-variables used for latent class analysis, alongside the number and size of latent classes, the goodness-of-fit index indicated a better fit with increasing sample size. This study was conducted after obtaining approval from the IRB of the author’s affiliated institution (KHSIRB-21-058(RA)). 

### 2.5. Data Analysis

For data analysis, SPSS for Windows (ver. 21.0, SPSS Korea Data Solution Inc., Seoul, Korea) and Mplus 8.6 (Muthen & Muthen, Los Angeles, CA, USA) were used. Details of the analysis methods are described as follows: for the participants’ personal, work-related, and health-related characteristics, frequency and percentage were calculated; for their severity of DCS symptoms, the minimum, maximum, mean, and standard deviation were calculated for each symptom; for the latent class types of participants’ DCS symptoms, latent profile analysis was performed using Mplus 8.6. Each variable was converted into a standardized score (*Z*-score), and, in order to determine the optimal number of latent profiles for the statistical criteria considering all the aspects of information index, the model comparison test, quality of classification, distribution rate, and interpretability were determined as follows: to test the model fit, information indices, namely, Akaike information criterion (AIC), Bayesian information criterion (BIC), and sample size-adjusted BIC (saBIC) were used. Lower values of AIC, BIC, and saBIC indicate greater suitability of the model. For model comparison, the Lo–Mendell–Rubin adjusted likelihood ratio test (LMR-LRT) and bootstrap likelihood ratio test (BLRT) were used; when the significance level of the LMR-LRT and BLRT was less than 0.05, the model was determined to be adequate. Entropy was used for the quality of classification, and classification was determined to be suitable if the entropy value was in the range of 0.8–1.0. As for the distribution rate, the values were different between previous studies, and there is no established criterion for determining the number of latent profiles. However, comparison by class is possible when each of the classes accounts for no less than 10% of the sample size; thus, a model with a distribution rate of all classes at 10% or higher was selected as a suitable one. The differences in the participants’ characteristics by latent class type were analyzed using the chi-square test, and variables with statistically significant differences were selected as the final ones for analysis through multinomial logistic regression. Multinomial logistic regression was performed to identify factors related to the classified latent class types.

## 3. Results

### 3.1. General Characteristics

Table 1 outlines the participants’ characteristics.

### 3.2. Severity of DCS Symptoms

The participants’ severity of DCS is described in this section. Among the musculoskeletal symptoms, the average score for joint pain was 5.36 ± 3.18. The overall average score of skin symptoms was 6.15 ± 6.50; as for the sub-symptoms, skin pain (2.17 ± 2.53) accounted for the highest proportion, followed by itching (2.06 ± 2.42) and rashes (1.92 ± 2.45). The finger edema symptom showed an average score of 2.95 ± 2.22. The overall average score of the central nervous system symptoms was 11.35 ± 8.77, and, for the sub-symptoms, headache (3.73 ± 2.78) accounted for the highest ratio, followed by numbness (3.28 ± 2.56), sensory changes (2.92 ± 2.55), seizures (0.93 ± 0.90), and unconsciousness (0.47 ± 0.74). The overall average score of inner ear symptoms was 21.03 ± 13.30, which was the highest of all DCS symptoms, and the sub-symptoms were in the order of dizziness (4.39 ± 2.72), hearing loss (4.33 ± 2.74), nausea (4.13 ± 2.84), tinnitus (4.13 ± 2.64), and vomiting (4.04 ± 2.73). The overall average score of chest symptoms was 4.27 ± 4.29, with cough (2.18 ± 2.47) and chest pain (2.09 ± 4.46) as sub-symptoms.

### 3.3. Latent Class Types of DCS Symptoms

In order to determine latent class types according to the response pattern for DCS symptoms, a latent profile analysis was performed, and the results on the fit indices of the latent classes are presented in Table 2. The names of each latent class were determined as follows: for the first class, it was the group showing high severity of symptoms corresponding to type 1 DCS; for the second class, it was the group showing high severity of symptoms corresponding to type 2 DCS; for the third class, it was the group showing mixed distribution of symptoms corresponding to type 1 and type 2 DCS. Therefore, the first class was named ‘type 1 symptoms group’, the second class was named ‘type 2 symptoms group’, and the third class was named ‘mixed symptoms group’.

### 3.4. Difference in Characteristics by Latent Class Type

Table 3 presents the difference in characteristics by latent class type. For personal characteristics, age (χ^2^ = 40.31, *p* < 0.001) and education level (χ^2^ = 28.15, *p* < 0.001) showed a significant difference by latent class type. For work-related characteristics, diving experience (χ^2^ = 29.99, *p* < 0.001) and break time (χ^2^ = 9.32, *p* = 0.011) showed a significant difference by latent class type. In terms of the health-related characteristics, menopausal period (χ^2^ = 40.10, *p* < 0.001), BMI (χ^2^ = 14.80, *p* = 0.013), and fatigue level (χ^2^ = 58.23, *p* < 0.001) showed a significant difference by latent class type.

### 3.5. Factors Related to Latent Class Types

The result of the model fit test showed significance, and the explanatory power of the model was 36% for Cox and Snell *R^2^*, and 40% for Nagelkerke *R*^2^ (Table 4). As a result of analysis by setting the ‘type 1 symptoms group’, which does not include high-severity DCS symptoms of type 2 DCS, as the reference group, the likelihood of being included in ‘type 2 symptoms group’ was higher by 3.35 times (95% CI = 1.27–8.85) and 5.18 times (95% CI = 1.88–14.30) for age groups of 60–69 and over 70, respectively, compared to those under 50. In addition, the likelihood was 2.36 times (95% CI = 1.25–4.11) higher in the group with the break time of 30 min or less than in that with a break time of more than 30 min. The likelihood was 3.17 times (95% CI = 1.73–5.83) higher in the group with a menopausal period of 21–30 years than in that with a period of 10 years or less. As for BMI, the likelihood was 2.10 times (95% CI = 1.05–4.19) higher in the group at the obesity stage than in the normal group. As for fatigue level, the likelihood was 8.52 times (95% CI = 3.86–20.74) higher in the group with a fatigue score of seven points or higher than in the group with a score of 0–3 points. The likelihood of being included in the ‘mixed symptoms group’ rather than the ‘type 1 symptoms group’ was 4.11 times (95% CI = 2.21–7.64) higher and 7.46 times (95% CI = 3.12–17.86) higher in the group with a menopausal period of 21–30 years and over 30 years, respectively, than in the group with a menopausal period of 10 years or less. In terms of BMI, the likelihood was 1.82 times (95% CI = 1.01–3.26) higher for the pre-obesity stage group than for the normal group. Regarding fatigue level, the likelihood was 2.12 times (95% CI = 1.34–4.36) higher for the group with a fatigue score at 4–6 points than for that with a fatigue score at 0–3 points.

## 4. Discussion

In this study, according to the participants’ response patterns to the DCS symptoms, three latent class types of the symptoms were identified. Examining previous studies related to DCS symptom types, Golding and his colleagues [35] classified DCS into type 1 DCS and type 2 DCS according to clinical manifestations, and Vann [4] also reported the classification into type 1 and type 2 DCS. In most cases, DCS is classified into type 1 and type 2 only in clinical practice, and, when both types are present, it is classified as type 2 DCS with high severity [4]. However, in DCS, multiple symptoms are manifested simultaneously, requiring a comprehensive examination of the symptoms that the individuals complain of. Since symptoms from nursing perspectives comprise highly subjective evaluations, and individuals’ levels of discomfort in daily lives differ accordingly, simply classifying DCS into type 1 and type 2 has limitations in terms of managing these symptoms. However, this study evaluated the severity of DCS symptoms perceived by the participants, as well as the presence/absence of the symptoms, allowing classification of latent class types of DCS symptoms into the ‘Type 1 symptoms group’, ‘type 2 symptoms group’, and ‘mixed symptoms group’.

As a result of this study, the ‘type 2 symptoms group’ was identified as one of the latent class types of DCS symptoms in Jeju haenyeos, with severe symptoms corresponding to type 2 DCS. It was identified as a group representing haenyeos of older age who started fishery diving when they were young, with longer diving experience. Unlike the other latent classes, the ‘type 2 symptoms group’ showed clear manifestation of neurological symptoms among DCS symptoms; thus, it is suggested that this group requires more active management and intervention by physicians and nurses. In a previous study on divers, it was reported that symptoms of the central nervous system were the most representative ones among those corresponding to type 2 DCS [36]; similarly, in this study, among the DCS symptoms, participants reported the highest severity of central nervous system symptoms. This can be explained by the etiology of DCS onset. When the ambient pressure is lowered by diving, nitrogen in the body becomes supersaturated, thus obstructing blood flow and causing DCS. In particular, nitrogen solubility has been shown to be higher in fatty tissues, and lipids comprise the major portion of the central nervous system, indicating a high risk of DCS [37]. Therefore, on the industrial level, the ‘type 2 symptoms group’ needs to be screened by health professionals, with the establishment of a system for haenyeos in which symptoms of the central nervous system are differentiated from the general symptoms of aging; when symptoms are physically manifested, haenyeos should be able to recognize them, asking for the assistance of health professionals or family members to enable their early management.

Next, the ‘mixed symptoms group’ was identified as one of the latent class types of DCS symptoms in Jeju haenyeos, showing mixed manifestations of type 1 and type 2 DCS symptoms. The largest number of participants in this study was classified into the ‘mixed symptoms group’ compared to the other two latent classes. In general clinical practice, when both types of DCS symptoms are manifested, this is mostly classified as type 2 DCS only [13]. There have been limited prior studies that showed approaches with classification other than just type 1 and type 2 for DCS symptoms; thus, it is difficult to make direct comparisons with the results of this study. However, setting the assessment of symptoms perceived by individuals as a reference, it can be inferred that, for DCS in Jeju haenyeos, a high proportion of the participants exhibited mixed symptoms of Type 1 and Type 2 DCS. In current clinical practice, DCS is classified into type 1 and type 2 only; however, on the basis of this study’s results, in the ‘mixed symptoms group’, although there were mixed manifestations of both type 1 and type 2 symptoms, type 2 symptoms were assessed with mild severity, which may lead to the participants not taking type 2 DCS-related symptoms seriously, and not paying proper attention for their management. However, when these types of patients can be identified and managed from an early stage, it is thought to prevent further aggravation of type 2 symptoms.

In this study, age, break time, menopausal period, BMI, and fatigue level were identified as factors of high correlation with the ‘type 2 symptoms group’. Through the results, it was interpreted that the ‘type 2 symptoms group’ consists of haenyeos who are older and have a longer experience in fishery diving compared to those in the other latent classes. This result is consistent with a previous study, which reported that structural and functional alterations of blood vessel walls with advancing age increase individuals’ sensitivity to DCS; thus, older age is associated with higher severity of DCS [25]. Another study reported that postmenopausal women undergo degenerative changes in their bones due to hormonal changes, leading to difficulties in diving and the occurrence of DCS [37], alongside a report on the increased risk of DCS due to an increase in women’s body fat, since it leads to higher nitrogen solubility [38]. The ‘type 2 symptoms group’ is a high-risk group with clear manifestations of neurological symptoms that require management by physicians and doctors, and, for this group, the key priority is the management of symptoms to prevent further aggravation. However, in practice, there is a lack of safety and health education for haenyeos, and continuous education, training, and management of DCS are required through health professionals such as nurses. In addition, it is thought that programs providing information to increase accessibility to local community resources for obesity management for Jeju haenyeos, and the development of programs that can be carried out with families at home would be highly useful for haenyeo groups that have many aged women divers.

For the ‘mixed symptoms group’, menopausal period, BMI, and fatigue level were identified as factors of high correlation. In this study, it can be seen that, for the ‘mixed symptoms group’, there was a high proportion of women with a long menopausal period compared to those in their early stage of menopause; the relationship between menopausal period and DCS occurrence in haenyeos needs to be investigated from the perspectives of hormonal or metabolic changes in women [39]. In addition, since the ‘mixed symptoms group’ identified in this study was not investigated or discussed in previous studies, it is difficult to make accurate comparisons. However, considering the level of obesity and fatigue in the ‘mixed symptoms group’, it is thought that preventive healthcare is important for haenyeos in this group, requiring active management. To this end, it is necessary to comprehensively examine the health management programs currently applied in Jeju Province, restructure the programs that were effective for the management of obesity and fatigue tailored to Jeju haenyeos, and apply these programs to the industry.

This study had some limitations. Since this study was conducted on some haenyeos in the Jeju region, it was not possible to consider all differences in their work environment according to regional characteristics within the Jeju Province. In addition, factors such as age and menopause as influencing factors on the DCS of haenyeos require careful interpretation, and it is necessary to confirm the relationship through repeated research. Therefore, further research is needed regarding DCS symptoms for haenyeos in various regions. Furthermore, this study was a cross-sectional survey research; if the changes in DCS symptoms and latent class types over time can be investigated, a systematic approach can be provided for the health management of Jeju haenyeos.

## 5. Conclusions

The symptoms of DCS in Jeju haenyeos were categorized into latent classes, and the types and characteristics of the latent classes were investigated. The results provided basic data for further DCS-related research with Jeju haenyeos. As opposed to existing studies in which DCS symptoms were simply classified into type 1 and type 2, the latent classes of DCS symptoms in Jeju haenyeos were categorized into three types, and the characteristics for each latent class were identified. The findings of this study are expected to contribute to establishing specific and practical solutions for the prevention of DCS symptoms, thus providing a basis for the health management of Jeju haenyeos. In future studies, it is necessary to confirm the DCS in consideration of the subject’s demographic or health-related characteristics. In order to reduce decompression sickness of haenyeos, repeated and systematic education is necessary during initial diving training.

## Figures and Tables

**Table 1:** General characteristics.

Variables	Categories	*n*	%	M ± SD
**Personal characteristics**				
Age (years)	<50	36	6.8	65.64 ± 10.85
	50–59	152	28.8	
	60–69	205	38.9	
	≥70	134	25.5	
Personal income (10,000 KWW)	<100	122	23.2	150.10 ± 60.82
	100–149	157	29.8	
	150–199	123	23.3	
	≥200	125	23.7	
Education level	Uneducated	105	19.9	
	Elementary-school graduate	166	31.5	
	Middle-school graduate	130	24.7	
	High-school graduate or higher	126	23.9	
**Work-related characteristics**				
Diving class	Prize	194	36.8	
	Middle	173	32.8	
	Under	160	30.4	
Diving experience (years)	≤30	108	20.5	41.31 ± 16.56
	31–40	100	19.0	
	41–50	179	34.0	
	>50	140	26.5	
Diving skill acquisition path	Educational institution	42	8.0	
	Senior haenyeo	313	59.4	
	Other (non-haenyeo)	172	32.6	
Diving-related education	Yes	84	15.9	
	No	443	84.1	
Diving soaring experience	Yes	61	11.6	
	No	466	88.4	
Surface break time (min)	≤1	218	41.4	1.74 ± 0.74
	>1	309	58.6	
Break time (min)	≤30	310	58.8	30.59 ± 19.75
	>30	217	41.2	
**Health-related characteristics**				
Medical history within the last 2 years (number of diagnoses)	≤1	98	18.6	2.19 ± 1.49
	2–3	305	57.9	
	>3	124	23.5	
Menopausal period (years)	≤10	132	25.0	18.96 ± 10.01
	11–20	178	33.8	
	21–30	157	29.8	
	>30	60	11.4	
Body mass index (BMI)	Normal	159	30.2	
	Pre-obesity	226	42.9	
	Obesity stage	142	26.9	
Fatigue level	0–3 (mild)	95	18.0	5.37 ± 1.98
	4–6 (moderate)	255	48.4	
	≥–7 (severe)	177	33.6	

M = mean, SD = standard deviation.

**Table 2:** Model fit indices of the latent profile analysis.

Standards of Classification		Number of Latent Profiles			
		1	2	3	4
**Information index**	AIC	8991.36	7549.86	6587.29	6272.25
	BIC	9042.57	7630.93	6698.23	6413.07
	saBIC	9004.48	7570.62	6615.70	6308.32
**Model comparison test**	LMR-LRT	-	1423.07	954.80	321.70
	LMR-LRT (*p*)	-	<0.001	<0.001	<0.001
	BLRT (*p*)	-	<0.001	<0.001	<0.001
**Quality**	Entropy	-	0.97	0.98	0.98
**Distribution rate (%)**	1	100	64.04	26.00	10.17
	2		35.96	33.57	37.78
	3			40.43	25.70
	4				26.35

AIC = Akaike information criterion, BIC = Bayesian information criterion, saBIC = sample size-adjusted BIC, LMR-LRT = Lo–Mendell–Rubin adjusted likelihood ratio test, BLRT = bootstrap likelihood ratio test.

**Table 3:** Differences in characteristics by latent profiles.

Variables	Categories	Type 1 Symptoms Group (*n* = 137)	Type 2 Symptoms Group (*n* = 178)	Mixed Symptoms Group (*n* = 212)	χ^2^	*p*
		*n* (%)	*n* (%)	*n* (%)		
**Personal characteristics**						
Age (years)	<50	15 (2.8)	7 (1.3)	14 (2.7)	40.31	<0.001
	50–59	52 (9.9)	41 (7.8)	59 (11.2)		
	60–69	46 (8.7)	72 (13.7)	87 (16.5)		
	≥70	24 (4.6)	58 (11.0)	52 (9.8)		
Personal income (10,000 KRW)	<100	32 (6.1)	43 (8.2)	47 (8.9)	12.37	0.182
	100–149	51 (9.7)	50 (9.5)	56 (10.6)		
	150–199	25 (4.7)	53 (10.1)	45 (8.5)		
	≥200	29 (5.5)	32 (6.1)	64 (12.1)		
Education level	Uneducated	23 (4.4)	30 (5.7)	52 (9.9)	28.15	<0.001
	Elementary-school graduate	37 (7.0)	73 (13.9)	56 (10.6)		
	Middle-school graduate	31 (5.9)	32 (6.1)	67 (12.7)		
	High-school graduateor higher	46 (8.7)	43 (8.2)	37 (7.0)		
**Work-related characteristics**						
Diving class	Prize	47 (8.9)	72 (13.7)	75 (14.2)	2.63	0.615
	Middle	50 (9.5)	56 (10.6)	67 (12.7)		
	Under	40 (7.6)	50 (9.5)	70 (13.3)		
Diving experience (years)	≤30	35 (6.6)	39 (7.4)	34 (6.5)	29.99	<0.001
	31–40	43 (8.2)	26 (4.9)	31 (5.9)		
	41–50	32 (6.1)	60 (11.4)	87 (16.5)		
	>50	27 (5.1)	53 (10.1)	60 (11.4)		
Diving skill acquisition path	Educational institution	17 (3.2)	14 (2.7)	11 (2.1)	12.12	0.062
	Senior haenyeo	68 (12.9)	114 (21.6)	131 (24.9)		
	Other (non-haenyeo)	52 (9.9)	50 (9.5)	70 (13.3)		
Diving-related education	Yes	25 (4.7)	27 (5.1)	32 (6.1)	0.74	0.681
	No	112 (21.3)	151 (28.7)	180 (34.2)		
Diving soaring experience	Yes	21 (4.0)	15 (2.8)	25 (4.7)	3.62	0.164
	No	116 (22.0)	163 (30.9)	187 (35.5)		
Surface break time (min)	≤1	60 (11.4)	72 (13.7)	86 (16.3)	0.45	0.804
	>1	77 (14.6)	106 (20.1)	126 (23.9)		
Break time (min)	≤30	89 (16.9)	90 (17.1)	131 (24.9)	9.32	0.011
	>30	48 (9.1)	88 (16.7)	81 (15.4)		
**Health-related characteristics**						
Medical history within the last 2 years (number of diagnoses)	≤1	16 (3.0)	37 (7.0)	45 (8.5)	7.15	0.130
	2–3	90 (17.1)	96 (18.2)	119 (22.6)		
	>3	31 (5.9)	45 (8.5)	48 (9.1)		
Menopausal period (years)	≤10	55 (10.4)	42 (8.0)	35 (6.6)	40.10	<0.001
	11–20	48 (9.1)	59 (11.2)	71 (13.5)		
	21–30	26 (4.9)	63 (12.0)	68 (12.9)		
	>30	8 (1.5)	14 (2.7)	38 (7.2)		
Body mass index (BMI)	Normal	46 (8.7)	54 (10.2)	59 (11.2)	14.80	0.013
	Pre-obesity	61 (11.6)	60 (11.4)	105 (19.9)		
	Obesity stage	30 (5.7)	64 (12.1)	48 (9.1)		
Fatigue level	0–3 (Mild)	26 (4.9)	11 (2.1)	58 (11.0)	58.23	<0.001
	4–6 (Moderate)	88 (16.7)	80 (15.2)	87 (16.5)		
	≥7 (Severe)	23 (4.4)	87 (16.5)	67 (12.7)		

**Table 4:** Factors related to latent profile type.

Group	Type 2 Symptoms Group						Mixed Symptoms Group					
Related Factors	B	SE	OR	*p*	95% CI		B	SE	OR	*p*	95% CI	
					Lower	Upper					Lower	Upper
**Personal characteristics**												
**Age (years)**												
<50	1						1					
50–59	0.52	0.50	1.70	0.301	0.63	4.53	−0.52	0.44	0.60	0.241	0.25	1.41
60–69	1.21	0.50	3.35	0.022	1.27	8.85	0.71	0.41	2.03	0.094	0.90	4.56
≥70	1.65	0.52	5.18	<0.001	1.88	14.30	1.30	0.44	3.66	0.053	1.55	8.60
**Education level**												
Uneducated	1						1					
Elementary-school graduate	0.47	0.44	1.60	0.283	0.68	3.75	−0.06	0.42	0.94	0.884	0.41	2.12
Middle-school graduate	−0.28	0.48	0.76	0.572	0.29	1.95	0.30	0.44	1.36	0.491	0.57	3.21
High-school graduate or higher	0.87	0.63	2.38	0.173	0.69	8.16	0.96	0.62	2.60	0.123	0.77	8.77
**Work-related characteristics**												
**Diving experience (years)**												
≤30	1						1					
31–40	−2.13	0.51	0.42	0.051	0.04	0.32	−1.77	0.52	0.17	0.063	0.06	0.48
41–50	−0.58	0.56	0.56	0.302	0.19	1.68	−0.19	0.56	0.83	0.731	0.28	2.46
>50	−0.25	0.60	0.78	0.673	0.24	2.51	−0.31	0.59	0.74	0.601	0.23	2.34
**Break time (min)**												
>30	1						1					
≤30	0.86	0.28	2.36	<0.001	1.35	4.11	0.45	0.28	1.57	0.112	0.91	2.70
**Health-related characteristics**												
**Menopausal period (years)**												
≤10	1						1					
11–20	0.48	0.28	1.61	0.090	0.93	2.80	0.84	0.29	2.32	0.092	0.87	7.68
21–30	1.16	0.31	3.17	<0.001	1.73	5.83	1.41	0.32	4.11	<0.001	2.21	7.64
>30	0.83	0.49	2.29	0.092	0.88	5.97	2.01	0.45	7.46	<0.001	3.12	17.86
**Body mass index (BMI)**												
Normal	1						1					
Pre-obesity	−0.07	0.32	0.93	0.834	0.50	1.73	0.60	0.30	1.82	0.041	1.01	3.26
Obesity stage	0.74	0.35	2.10	0.041	1.05	4.19	0.25	0.35	1.28	0.492	0.64	2.54
**Fatigue level**												
0–3 (Mild)	1						1					
4–6 (Moderate)	0.66	0.43	1.93	0.132	0.83	4.48	2.84	0.70	2.12	0.042	1.04	4.36
7 (Severe)	2.92	0.50	8.52	<0.001	3.86	20.74	0.74	0.42	2.09	0.083	0.91	4.77

B = standard regression, SE = standard error, OR = odds ratio, 95% CI = 95% confidential interval, −2 log likelihood = 783.52, χ^2^ = 231.21, df = 34, *p* < 0.05, Cox and Snell *R*^2^ = 0.36, Nagelkerke *R*^2^ = 0.40, Reference group = type 1 symptoms group; 1 = reference.

## Data Availability

The data presented in this study are available on request from the corresponding author.

## References

[1] Yoon D.H., Yang K.M., Oh H.G., Kim M., Chang M. (2021). Consumption changes during COVID-19 through the Analysis of credit card usage: Focused on Jeju province. J. Econ. Bus Manag..

[2] Park J.H., Lee J.Y., Cha S.W., Shin S.R., Kim S.J. (2018). Daily variation in body temperature and heart rate of breath-holding women divers ‘Haenyeo’ in Jeju Island. J. Korean Soc. Environ..

[3] Sánchez-Villalobos J.M., Fortuna-Alcaraz M.L., Serrano-Velasco L., Pujante-Escudero Á., Garnés-Sánchez C.M., Pérez-Garcilazo J.E., Olea-González A., Pérez-Vicente J.A. (2022). Breath-Hold Diving-Related Decompression Sickness with Brain Involvement: From Neuroimaging to Pathophysiology. Tomography.

[4] Mitchell S.J., Bennett M.H., Moon R.E. (2022). Decompression Sickness and Arterial Gas Embolism. N. Engl. J. Med..

[5] Lundell R.V., Arola O., Suvilehto J., Kuokkanen J., Valtonen M., Räisänen-Sokolowski A.K. (2019). Decompression illness (DCI) in Finland 1999–2018: Special emphasis on technical diving. Diving Hyperb. Med..

[6] DeGorordo A., Vallejo-Manzur F., Chanin K., Varon J. (2003). Diving emergencies. Resuscitation.

[7] James P.B., Jain K.K. (2009). Decompression Sickness: Textbook of Hyperbaric Medicine.

[8] Kim J.I., Kim M. (2018). Health care experiences of Korean women divers (Jeju haenyeos). Qual. Health Res..

[9] Grünig H., Nikolaidis P.T., Moon R.E., Knechtle B. (2017). Diagnosis of swimming induced pulmonary edema. Front. Physiol..

[10] Oh K.J., Park J.Y., Sa K.J. (2008). Diving patterns and decompression sickness related symptoms of SCUBA divers. Korean J. Sports Med..

[11] Hafner M., van Stolk C., Saunders C., Krapels J., Baruch B. (2015). Health, Wellbeing and Productivity in the Workplace.

[12] Dos Santos T.M., Kozasa E.H., Carmagnani I.S., Tanaka L.H., Lacerda S.S., Nogueira-Martins L.A. (2016). Positive effects of a stress reduction program based on mindfulness meditation in Brazilian nursing 79 professionals: Qualitative and quantitative evaluation. J. Sci. Heal..

[13] Levett D.Z.H., Millar I.L. (2008). Bubble trouble: A review of diving physiology and disease. Postgrad. Med. J..

[14] Park H.C., Cho K.J. (2015). Correlation among knowledge of safety, compliance with safety rules, and ability to cope with emergency situations of scuba divers. Korean J. Emerg. Med. Ser..

[15] Kwon Y.T., Pyo H.G. (2015). A study on the safety measures proposed by diving accident type analysis. Korean J. Sport Sci..

[16] Kang E.S., Lim H.S., Kang J.H., Mun Y.J. (2009). The analysis on the causes of non-activating precautionary decompression stop by scuba divers. Korean J. Sport Sci..

[17] Kang K.S., Uhm D.C., Baek H.S. (2011). Factors Influencing on Safety knowledge of Scuba divers. Korea Acad. Ind. Coop. Soc..

[18] Neuman T.S. (1997). Pulmonary Disorders: Diving Medicine.

[19] Bove A.A. (1997). Cardiovascular Disorders: Diving Medicine.

[20] Boussuges A., Retali G., Bodéré-Melin M., Gardette B., Carturan D. (2009). Gender Differences in Circulating Bubble Production after SCUBA diving. Physiol. Funct. Imaging..

[21] Gempp E., Demaistre S., Louge P. (2014). Hypertension is predictive of recurrent immersion pulmonary edema in scuba divers. Int. J. Cardiol..

[22] Sa K.J. (2006). Incidence, Prevalence, and Prevention of Diving Related Disease of Diving Fishermen and Breath-Hold Divers in Korea.

[23] Cha S.G., Byun Y.S., Jeon M.J., Sakong J. (2019). Diving patterns and decompression sickness among South Korean fishery divers. J. Occup. Health..

[24] Lee W.D., Kim S.G., Kim M.H., Lee J.H. (2019). Air diving operation, management and planning for safe and effective underwater works. J. Korean Soc. Saf..

[25] Choi J.C., Lee J.S., Kang S.Y., Kang J.H., Bae J.M. (2008). Chronic daily headache with analgesics overuse in professional women breath-hold divers. J. Headache Pain..

[26] Kim W.J. (2005). Recompression therapy in decompression sickness. Cheju J. Med..

[27] Ko E.S., Kang K.J. (2018). Pain, depression, and health-related quality of life of older haenyeos (women divers). Asian Pac. Isl. Nurs. J..

[28] Hwang H.S., Choi H.R. (2003). The effect of breath hold diving on bone mineral density of women fishery diver. Ann. Occup. Environ Med..

[29] Jain K.K. (1999). Textbook of Hyperbaric Medicine.

[30] Park J.H. (2004). The Culture of Women in Jeju: With a Focus on Family and Married Life Jeju.

[31] Korea Occupational Safety and Health Agency Diver Health Care Guidelines. www.kosha.or.kr.

[32] Korean Society for the Study of Obesity Guideline for the Management of Obesity in Korea. http://general.kosso.or.kr.

[33] Lim E.J., Jang E.S., Son C.G. (2020). Characteristics of chronic fatigue syndrome (cfs): Based on qualitative content-analysis of patient description. Korean Public Health Res..

[34] Nylund K.L., Asparouhov T., Muthén B.O. (2007). Deciding on the number of classes in latent class analysis and growth mixture modeling: A monte carlo simulation study. Struct. Equ. Modeling.

[35] Golding F.C., Griffiths P., Hempleman H.V., Paton W.D.M., Walder D.N. (1960). Decompression sickness during construction of the Dartford Tunnel. Occup. Environ. Med..

[36] Neuman T.S. (2002). Arterial gas embolism and decompression sickness. Physiology.

[37] Tintinalli J.E. (2004). Emergency Medicine: A Comprehensive Study Guide.

[38] Gustavsson L.L., Hultcrantz E. (1999). Medical Aspect of Diving-A Sport for Both Women and Men. Lakarfidningen.

[39] Germonpré P., Balestra C. (2017). Preconditioning to reduce decompression stress in scuba divers. Aerosp. Med. Hum. Perform..

